# The evaluation of sexual dysfunction in male patients with migraine and tension type headache

**DOI:** 10.1186/1129-2377-14-46

**Published:** 2013-05-29

**Authors:** Durdane Aksoy, Volkan Solmaz, Betul Cevik, Yusuf Gencten, Fikret Erdemir, Semiha Gulsum Kurt

**Affiliations:** 1 Department of Neurology, Gaziosmanpasa University Faculty of Medicine, Tokat, Turkey; 2 Department of Urology, Gaziosmanpasa University Faculty of Medicine, Tokat, Turkey

**Keywords:** Migraine, Tension type headache, Erectile dysfunction, International Index of Erectile Function Scale

## Abstract

**Background:**

Erectile dysfunction (ED), defined as the inability to achieve or maintain an erection sufficient for satisfactory sexual performance, is a common condition. The psychological, hormonal, neurogenic and arterial pathologies, medications, chronic diseases have been reported in the etiology of the ED. This paper aims to study sexual dysfunction in the male patients with migraine and Tension type headache (TTH).

**Methods:**

30 migraine cases (Group M), 31 TTH cases (Group T) and 30 control cases (Group C) were included in the study. Patients were evaluated with medical history, physical examination, body mass index (BMI), Beck Depression Inventory, biochemical analysis and hormone profiles. ED was evaluated via International Index of Erectile Function Scale (IIEF). In statistical analysis, variant analysis, post-hoc tukey test, Pearson correlation test, *t*-test, and fisher's exact chi-square test were used.

**Results:**

The patients' mean age was 34.96+/−1.30, 35.54+/−1.52 and 32.26+/−1.38 for group M,T and C, respectively. There was no significant difference between the groups in terms of testosterone levels. Mean IIEF scores was 19.83+/−2.2, 20.39+/−1.35 and 27.83+/−0.34 in groups M,T,C. When M and T groups were compared with group C, there were significant differences, and there was no statistical difference when T and M groups were compared to each other. Beck Depression Scores were not significantly different in groups M, T and C.

**Conclusion:**

In this study, it was shown that, migraine and TTH affects the sexual functions negatively in male patients. Chronic diseases may cause sexual disorders in patients because of despair, guilt, and fear of death or pain. Our results suggest that, along with the effect of chronic disease and pain, there must be other complicated factors exist causing the development of SD in patients with migraine and TTH.

## Background

Erectile dysfunction (ED), defined as the inability to achieve or maintain an erection sufficient for satisfactory sexual performance [1], is a common condition, with a reported prevalence of 52% in men aged 40 to 70 years in the United States and 49% in men aged 50 to 80 years in Europe [2,3]. For normal penile erection the integration of normal psychological, neurological, and normal vascular processes are required. Several factors can disrupt the normal physiologic mechanisms involved in penile erection. In this context, the psychological, hormonal, neurogenic and arterial pathologies, medications, iatrogenic causes, and also systemic and chronic diseases have been reported in the etiology of the ED. Chronic diseases include diabetes mellitus, chronic renal failure, chronic obstructive lung disease, arthrosis, collagen tissue diseases, chronic hepatitis and other chronic infections, hypertension, multiple sclerosis and Behçet's disease [4-7].

Migraine is also a chronic disease and one of the most common causes of primary headache along with tension type headache (TTH). Sensitivity to stress factors, significant degradation in quality of life and workforce loss in communities are the common features of both headache types, and can noticeably affect social life in many situations. Sexual functions are also affected, and existence of sexual dysfunction can cause lack of self-confidence, social withdrawal, divorces and other social problems by the effect on the quality of life of both the patient and his partner [8,9]. In accordance with these findings, sexual dysfunction is noted in male cases of all other chronic diseases along with migraine [10].

This paper aims to study sexual dysfunction in the most common primary headache causes, TTH and migraine.

## Methods

Between January, 2012 and December, 2012, 30 migraine type headache cases (Group M), 31 tension type headache cases (Group T) and 30 control cases (Group C) were included in the study after informed consent was obtained. All individuals in the patient and control groups were married. The patients’ condition had been there for at least 6 months with no prophylactic treatment. Patients were evaluated with detailed medical history, physical examination, body mass index (BMI), routine hematologic and biochemical analysis, liver function tests and hormone profiles (Testosterone). ED was evaluated via International Index of Erectile Function Scale (IIEF). IIEF is one of the most commonly used tests for the investigation of patients with sexual dysfunction. In this test, answers receive points between 0 and 5. Erectile dysfunction is classified as severe ED (1–10 points), intermediate ED (11–16), intermediate-mild ED (17–21 points), mild ED (22–25 points) and non-erectile dysfunction (26–30) according to answers to 15 questions [11].

Patients with psychosis or similar psychiatric disorders, antidepressant, anticonvulsant, and antipsychotic drug use, chronic disorder, chronic pain, BMI greater than 30, chronic alcohol and tobacco use, history of pelvic operation, and metabolic, endocrine, or neurologic disorders were excluded from the study. All patients were analyzed with the Beck Depression Inventory for their psychiatric state. The Beck Depression Inventory was developed by Beck in 1961. It aims to evaluate the level of emotional, cognitive and motivational symptoms and has 21 questions. Each part of the test contains questions that express a behavioral pattern specific to depression, scaled from least to greatest. In all cases, headache and its classification were made by a neurology specialist according to International Headache Society (IHS) criteria. The hospital ethics committee approved the study, and written informed consent was obtained from each patient after the nature and purpose of the study was fully explained. All experiments were performed in accordance with the Declaration of Helsinki.

When presenting data, percentage and standard deviation were used. In statistical analysis, variant analysis, post-hoc tukey test, Pearson correlation test, *t*-test, and fisher's exact chi-square test were used. All statistical analyses were made with SPSS-15 software. All tests were found significant for p < 0.05.

## Results

The mean age of the patients was 34.96 ± 1.30 year, 35.54 ± 1.52 year and 32.26 ± 1.38 year for group M, group T and group C, respectively. There was no statistical significant difference in age distribution (p = 0,22). The average number of days with headache per month was 4.06 ± 3.35 in Group M and 3.36 ± 2.28 in group T. Mean BMI of group M, group T and group C were 24.68 ± 0.56, 27.06 ± 0.79, 25.85 ± 0.49 respectively. Total testosterone levels were 4.82 ± 0.37 in group M, 4.51 ± 0.34 in group T and 4.59 ± 0.30 in group C and there was no statistical difference between groups (Table 1). Mean IIEF scores was 19.83 ± 2.2, 20.39 ± 1.35 and 27.83 ± 0.34 in groups M,T, C. When M and T groups were compared with group C, there were significant differences between T and M groups and group C, but there was no statistical difference when T and M groups were compared to each other (Table 1). When TSH, T3, T4 and testosterone levels were compared between groups, we identified no significant difference. There was a negative correlation between age and IIEF. We found no correlation with headache duration, testosterone level in M and T groups.

**Table 1:** Age, BDS, IIEF and BMI scores, Testosterone levels in Migraine, TTH and control groups

	**Migraine**	**TTH**	**Control**	**F and p**
**Age (Year)**	34.96 ± 1.30	35.54 ± 1.52	32.26 ± 1.38	F = 1.53
				p = 0.22
**IIEF**	19.83 ± 2.2	20.39 ± 1.35	27.83 ± 0.34	F = 16.95 **p = 0.001**

**Testosterone**	4.82 ± 0.37	4.51 ± 0.34	4.59 ± 0.30	p = 0.37
**BMI (kg/m**^**2**^**)**	24.68 ± 0.56	27.06 ± 0.79	25.85 ± 0.49	p = 0.56
**BDS**	2.86 ± 3.31	4.06 ± 4.02	3.50 ± 4.14	p = 0,51

IIEF: International Index of Erectile Function, BMI: Body Mass Index, TTH: Tension type headache, BDS: Beck Depression Scores.

While 6.66% of patients in group M had IIEF values below 10, 60% of the patient values were between 11 and 22. 3.22% of patients in group T had IIEF values below 10, and 51.61% of the patients in this group had values between 17–22. In group M, there were no significant statistical differences between intermediate-severe sexual dysfunction groups and mild-intermediate sexual dysfunction groups in terms of total testosterone levels. Similarly, in group T, there were no significant statistical differences between intermediate-severe sexual dysfunction groups and mild-intermediate sexual dysfunction groups in terms of total testosterone levels (Table 2). Additionally, Beck Depression Scores were not significantly different in groups M, T and C (p = 0.51) (Table 1). Other socio-demographic characteristics of the groups were expressed in Table 3.

**Table 2:** International index of erectile dysfunction (IIEF) scores in groups

	**Migraine**		**Tension type**		**Control**	
**IIEF**	**n**	**%**	**n**	**%**	**n**	**%**
**No sexual dysfunction (23–30)**	10	33.33	14	45.16	30	100
**Mild-Intermediate Sexual Dysfunction (11–22)**	18	60.00	16	51.61	0	0
**Severe Sexual Dysfunction (≤10)**	2	6.66	1	3.22	0	0
**Total**	30	100	31	100	30	100

**Table 3:** Comparison of research groups according to sociodemographic factors

**Features**		**Migraine**		**Tension type**		**Control**		
		**n**	**%**	**n**	**%**	**n**	**%**	
**Education**	Primary school	17	56.6	20	64.5	15	50	*x*^2^ = 0.73; p = 0.25
	High school and above	13	43,3	11	35,5	15	50	
**Period of disease**	0-5 year	13	43.3	18	58.1	-	-	*x*^2^ = 2.12; p = 0.73
	6-10 year	13	43.3	8	25.8	-	-	
	10 and more	4	13.3	5	16.1	-	-	
**Income**	Intermediate	27	90.0	28	90.3	23	76,7	*x*^2^ = 2.99; p = 0.65
	Good	3	10.0	3	9.7	7	23.3	
**BMI (kg/m**^**2**^**)**	Normal (<24,9)	16	53.3	10	32.3	9	30.0	*x*^2^ = 4.22; p = 0.56
	Overweight and obese (≥25)	14	46.7	21	67.7	21	70.0	

## Discussion

Erectile dysfunction is an important health problem that increases in prevalence with advancing age. It negatively affects patients’ and their partners’ social lives and mental health. Erection is based on central, hormonal and peripheral factors. The primary event in erection is after sexual arousal, following relaxation of smooth muscles in the corpus cavernosum, and the filling of sinusoids with blood. As a result of cavernosal neural crest stimuli and parasympathetic stimuli from preganglionic neurons in the intermediolateral columns in S2-4, neurogenic and endothelial nitric oxide are released. This is followed by a reaction that contains L-arginine tetrahydrobiopterin. As a result of this reaction, lipophilic nitric oxide is produced and enters the cavernosal smooth muscle cells. cGMP decreases Ca^+2^ levels by different mechanisms, then initiates and mediates the erection by relaxing cavernosal smooth muscles. As understood, erection is a complex process including vascular structures, peripheral and central mediators and neural system [12-15].

As mentioned in the guidelines neurogenic, organic, vascular and endocrinologic etiologies can result in ED [16]. In addition, personal mental system and related psychogenic reasons also take part in the etiology of ED [17]. It is known that systemic and chronic disorders are included in ED etiology [18-20]. In this context, Sasaki et al. found a 90% prevalence of ED in a study that monitored 1118 cases with a diagnosis of Diabetes Mellitus (DM) [21]. Also Giuliano et al. from France found a 71% prevalence in a study of 7689 DM patients [22]. MS, a chronic systemic disease that affects young sexually active men, is reported in ED etiology and Thoma et al. found a 50-90% prevalence in a study with MS patients [23]. Headaches were investigated for their effects on sexual life in many studies. In Taiwan, 5362 ED patients were investigated for migraine, and compared to the control group it was found statistically increased especially in the 30–39 age range [24]. In another study, sexual dysfunction was found more often in patients with migraines, and pathogenesis was associated with serotonin [25]. In a study of Timothy et al. migraine was compared with tension type headache in terms of sexual dysfunction and it was found more often in the tension type headache group [26]. Our results were consistent with the literature: each headache group was found statistically significant for sexual dysfunction compared to control group. However there were no significant differences between group M and T.

Several mechanisms are suggested for the cause of ED via systemic and chronic disorders. According to this, primary chronic disease can affect the vascular structures and corpus cavernosum or cavernosal neurons that are important for erection. It is suggested that ED can be caused by Multiple Sclerosis (MS) or neurogenic diseases by affecting cavernosal and pelvic neurons, DM by effecting cavernosal smooth muscle or vascular muscle structures or peripheral neuropathy. It is also known that mood disorders caused by chronic diseases can cause ED. Treatment methods or psychological states of these systemic or chronic diseases can cause sexual dysfunction indirectly. In a study that contained 17 MS cases, psychiatric disorders were found in 12.56% [27] and in another study psychiatric disorders were found in 38% of patients with eosinophilic fasciitis [28]. Even though it is known that chronic diseases can cause psychiatric disorders by causing mood disorders, the number of studies that examine relations of these diseases with ED are limited. In a study that included 100 male cases of Brucellosis between ages 20–45, ED was found in 68% [29].

Chronic benign or malignant diseases or their treatments may cause sexual disorders in patients because of despair, desperation, guilt, and fear of death or pain. Also, during chronic diseases just like mental and physical energy, sexual energy correlates with survival or coping with chronic diseases [13]. Underlying mechanisms of chronic diseases such as migraine, irritable bowel syndrome, and interstitial cystitis were explained with different deregulations in central nervous system. It is suggested that this mechanism is related to the one that causes sexual dysfunction [24,26,30,31]. Current researches show that chronic pain activates much different mechanisms than acute pains. Therefore, complications with chronic pain might start a common set of processes in central nervous system and this complicated processes involves neurotransmitters, such as serotonin, dopamine [24]. It is possible that deregulations in dopamine pathways, which plays an important role especially in etiopathogenesis of migraine, affect the sexual functions negatively [24,30,31]. It is also suggested that, increase in the amount of serotonin (5-HT) in mid-brain can cause sexual dysfunction by having an antagonist effect on testosterone of male migraine patients [25,26]. The high rates of comorbidity between 'Primary headache associated with sexual activity' and migraine (25%), as well as TTH (45%) is also known [32]. Thus, the thought of triggering or increasing the headache during sexual activity, might be affecting the sexual performance of the patients negatively. In migraine cases, this disorder affects patients’ mental state and cause ED. The studies indicates that depression and the other psychiatric disorders, which are known to cause SD, can often accompany migraine and TTH [24,33]. However, compared to the control group, Beck Depression Scores were not significantly different in groups M and T in our study. This result suggests, comorbid psychiatric condition alone cannot explain the sexual function disorder in both types of headache.

## Conclusions

In our study, IIEF scores of patients with both migraine and tension type headache were significantly lower compared with the control group, and no direct relation of ED with Beck depression scores and BMI was established in these patients. These results suggest that, along with the effect of chronic disease and pain, there must be other complex and heterogeneous factors exist causing the development of SD in patients with migraine and TTH. As a result, prospective, randomized studies in large populations are required for revealing the etiology of sexual dysfunction in patients with headaches.

## Abbreviations

ED: Erectile dysfunction; TTH: Tension type headache; BMI: Body mass index; IIEF: International index of Erectile Function; Group M: Migraine group; Group T: Tension type headache group; Group C: Control group; DM: Diabetes Mellitus; MS: Multiple Sclerosis.

## Competing interest

The authors declare no potential conflicts of interests with respect to the authorship and/or publication of this article.

## Authors’ contributions

DA, VS, YG: have made substantial contributions to conception and design, acquisition of data, analysis and interpretation of data; FE, BC: have been involved in drafting the manuscript; SGK, FE: were involved in revising the manuscript critically for important intellectual content and have given final approval of the version to be published. All authors read and approved the final manuscript.
